# Differential Regulation of PDE5 Expression in Left and Right Ventricles of Feline Hypertrophy Models

**DOI:** 10.1371/journal.pone.0019922

**Published:** 2011-05-19

**Authors:** Xiaoyin Shan, Kenneth B. Margulies

**Affiliations:** Cardiovascular Institute, University of Pennsylvania School of Medicine, Philadelphia, Pennsylvania, United States of America; McMaster University, Canada

## Abstract

**Background:**

Though long known to affect smooth muscle biology, recent studies indicate that phosphodiesterase 5 (PDE5) is also expressed in myocardium. Recognizing that the regulation of PDE5 in hypertrophy is not well understood, we assessed the response of PDE5 expression and the level of cGMP-dependent kinase I (cGKI) in the left and right ventricles of feline hypertrophy models.

**Methodology/Principal Findings:**

Using a cDNA library of feline aortic smooth muscle cells, we identified and cloned PDE5 cDNA for the first time in this species. The sequence shares 98% identity with its human orthologue at the amino acid level. *E. coli* expression of the cloned allele allowed selection of antibodies with appropriate specificity, facilitating the analysis of PDE5 expression in feline models created by selective proximal aortic (Ao) or pulmonary artery (PA) banding that resulted in hypertrophy of the left ventricle (LV) and right ventricle (RV), respectively. We demonstrated that PDE5 expression responded differentially with a decreased expression in the LV and an increased expression in the RV in the Ao-banded model. Similarly, in the PA-banded model, LV showed reduced expression while the RV expression was unaltered. In addition, the expression of cGKI was significantly decreased in the RV of Ao-banded group, correlating inversely with the increase in PDE5 expression.

**Conclusions/Significance:**

The differential regulation of PDE5 and cGKI expression suggests that the mechanisms involved in hypertrophy could be different in RV vs. LV. Reciprocal PDE5 and cGKI expression in the RV of Ao-banded model suggests functional significance for PDE5 up-regulation.

## Introduction

PDE5 is a cGMP specific phosphodiesterase that converts cGMP to GMP. By hydrolyzing cGMP, PDE5 regulates intracellular cGMP levels that affect the tone of vascular smooth muscle, where PDE5 is abundantly expressed [1], [2]. Cyclic GMP exerts its functions through downstream effectors, including protein kinase G (PKG) and cyclic nucleotide-gated channels [3]. The PDE5 enzyme is a homodimer with an allosteric cGMP-binding site and a serine phosphorylation site in the N-terminal regulatory domain of each subunit. The binding of cGMP to the allosteric site activates PDE5 by increasing the binding affinity of cGMP to the catalytic site in the C-terminal catalytic domain. Phosphorylation of the serine residue by cGKI was shown to further enhance the substrate binding affinity to the catalytic site [4]. The regulation of PDE5 transcription is thought to be through sp-1 and AP2 transcription factors [5].

In animal models and patients with pulmonary hypertension, PDE5 levels in the smooth muscle are elevated, leading to reduced cellular cGMP concentrations that causes abnormal regulation of vasodilatory mechanisms [6]. Inhibition of PDE5 with sildenafil has been shown to reduce pulmonary vasoconstriction, thereby alleviating right ventricular overload and remodeling [7], [8], [9]. Although myocardial PDE5 expression has been reported in humans and other species, the function of myocardial PDE5 expression is not well understood [2]. PDE5 inhibitors have been used in a number of studies to discern the functions of the enzyme in cardiac hypertrophy and heart failure [10] [9], [11], [12], [13], [14], [15]. However, the results show that PDE5 inhibition is beneficial under certain conditions but not others [9], [12], [13], [14]. Differential regulation of cGMP/cGKI signaling pathway in the RV vs. LV under hypertrophic conditions is thought to affect the effectiveness of strategies to limit pathological myocardial remodeling through PDE5 inhibition [16].

Because PDE5 is critically involved in the regulation of cellular cGMP levels, determination of the abundance of PDE5 in RV and LV could provide further assessments of the cGMP/cGKI functions in RV and LV respectively in response to hypertrophic stimulations, since tissue PDE5 expression directly impacts the total cellular PDE5 activity. In this study, we identified and cloned PDE5 cDNA for the first time in feline. An antibody that recognizes the expressed protein was used to examine PDE5 expression levels in both the left and the right ventricles of two feline cardiac hypertrophy models relative to a normal control group. By comparing RV and LV PDE5 expression in response to the hypertrophic conditions, the existence of a chamber-dependent regulation could be assessed. Expression of cGKI, a downstream effector of cGMP, was also analyzed to further examine the impact of PDE5 on the cGMP signaling pathway.

## Results

### Identification and Isolation of a Feline cDNA Homologous to Human PDE5

**Figure 1:**
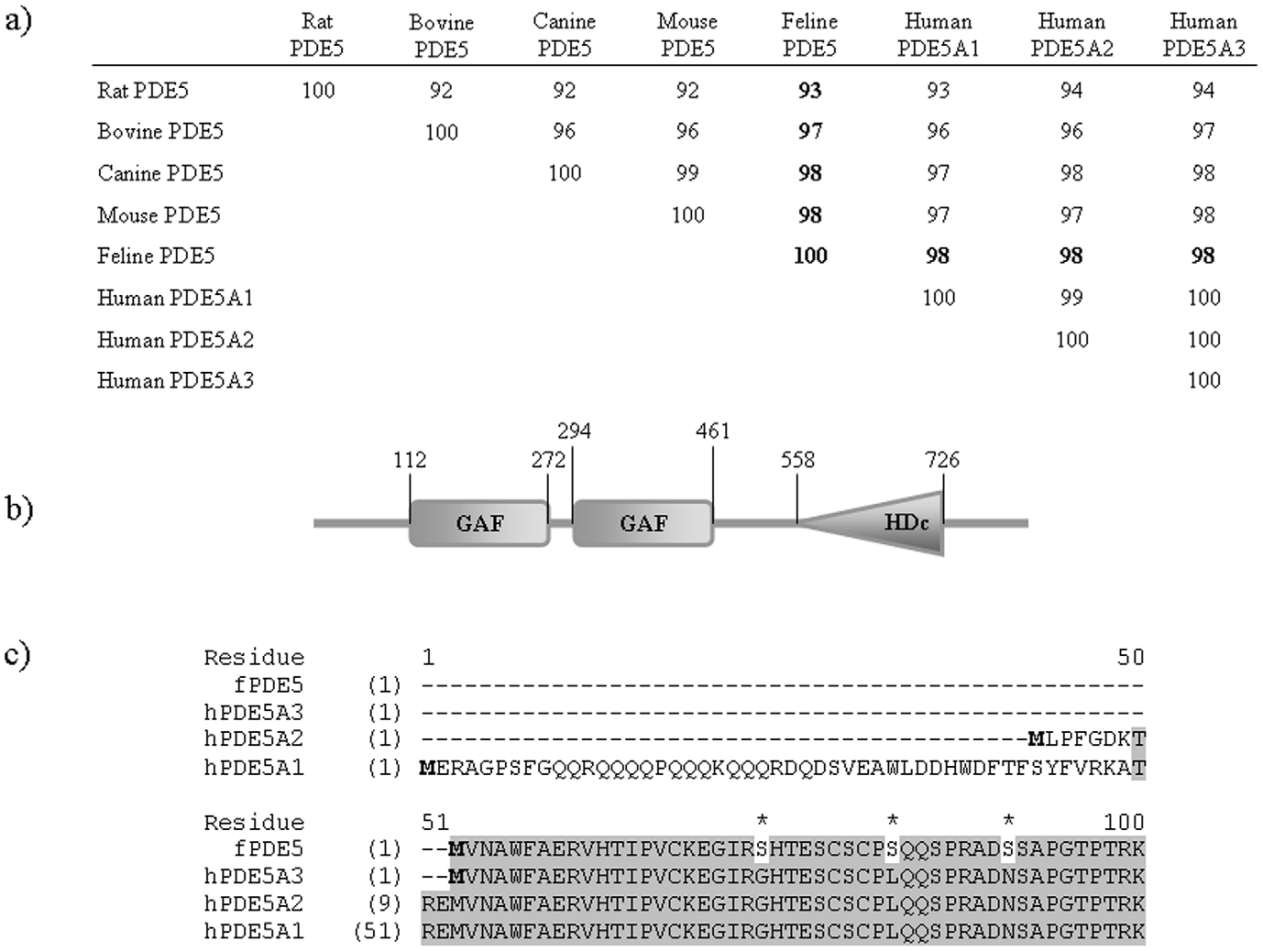
Sequence and Structural features of feline PDE5. (a) Similarity between feline PDE5 and PDE5s from other species. The feline PDE5 sequence was obtain from the translation of the cloned cDNA sequence. Sequences of PDE5 from other species were obtained from GenBank. Multi-sequence alignments were calculated using Clustal W. The % identities between feline and other species are in bold. (b) Domain structure of the feline PDE5 protein. GAF indicates cGMP binding domain, and HDc the phosphohydrolase catalytic domain. Structure prediction was performed using the SMART software. (c) Alignment of protein sequences from the N-terminal region of feline and human PDE5 variants. The shaded area indicates identical residues and the residues in feline PDE5 that are different from those of human variants are noted with asterisks. The start codons are in bold faces and amino acid residue numbers are indicated.

Feline hypertrophy models have been utilized to mimic disease states of human heart failure [17], [18]. However, the gene encoding PDE5 in feline was not previously identified. To accurately characterize the molecular mechanisms of PDE5 involvement in hypertrophy, the availability of the sequence information becomes important. Based on the current knowledge of PDE5 tissue distribution, we generated a cDNA library with cells isolated from aortic smooth muscle. Using primers hybridizing to the highly homologous regions of PDE5 among different species, we amplified DNA fragments from the cDNA library and sequenced these fragments. BLAST (Basic Local Alignment Search Tool [19]) analysis showed that these cDNA fragments were highly similar to human PDE5 cDNA. They correspond to a region of human PDE5 cDNA that began near the start codon and extended beyond the stop codon. By 5′-RACE, we were able to obtain the missing 5′- end of the feline cDNA and to assemble an ORF encoding a polypeptide of 824 amino acids. The nucleotide sequence was deposited at GenBank (HQ446880). Multiple sequence alignment results using Clustal W [20] showed that the feline ORF shared 98% identity with the human orthologue at the amino acid level, the highest among the known PDE5 sequences (Fig. 1a). Using the Simple Modular Architecture Research Tool [21], [22], the protein was predicted to contain both GAF and the catalytic domains (Fig. 1b). These domains are highly conserved among all the known PDE5s. At the N-terminus, the feline PDE5 ORF shared the highest similarity with splice variant 3 of human PDE5A (Fig. 1c).

### Detection of PDE5 Activities in Cell Extracts Expressing the Isolated cDNA Allele

**Figure 2:**
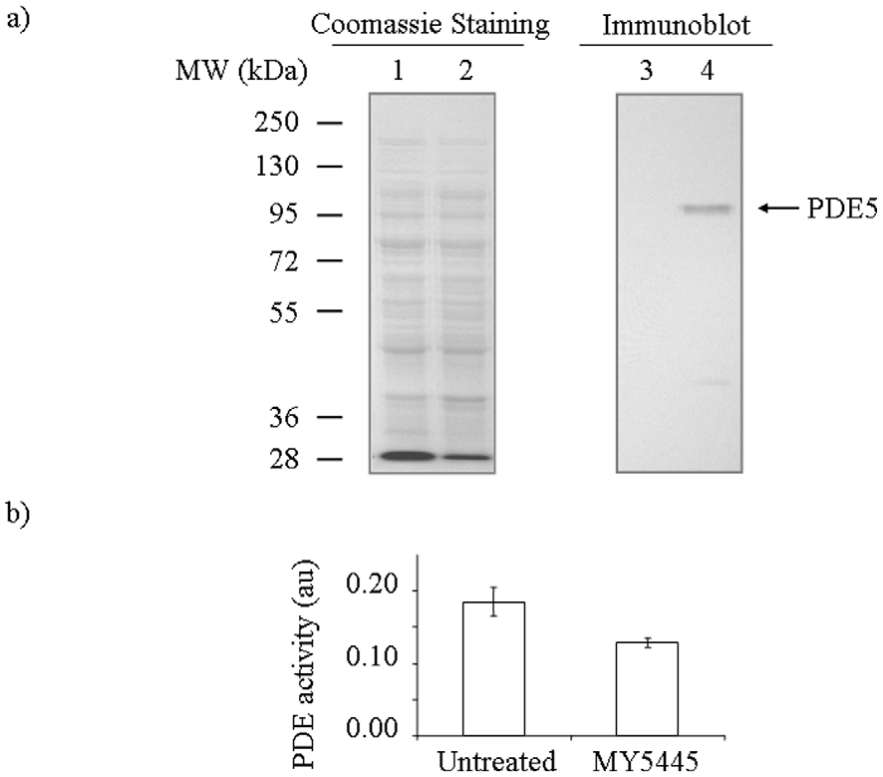
Immunoblotting and enzyme activity measurement of feline PDE5 expressed in E. coli. (a) Immunoblot analysis and coomassie blue staining of whole cell extracts made from *E. coli* cells carrying feline PDE5 expression plasmid pT7-PDE5 or a control vector pSNAP-tag(T7). Lanes 1 and 3 are extracts from cells carrying pSNAP-tag(T7). Lanes 2 and 4 are extracts from cells carrying pT7-PDE5. Lanes 1 and 2 are Coomassie blue staining of the gel. Lanes 3 and 4 are immunoblot using an anti-human PDE5 antibody. (b) Measurement of PDE5 activity. PDE activity of the extracts from cells carrying pT7-PDE5 in the presence or absence of a PDE5 inhibitor, MY5445, was shown (see Method for details). Basal level PDE activity determined with extract from cells carrying pSNAP-tag(T7) was subtracted. The error bars indicate standard deviation (n = 4).

To verify that the isolated cDNA encodes the feline PDE5, we cloned the entire ORF into pSNAP-tag(T7), under the control of T7 promoter, to obtain plasmid pPDE5-T7 that allows the expression of the cDNA in *E. coli*. After inducing the cultures of transformed BL21-AI strains with 0.1% arabinose, whole cell extracts were made from strains carrying either pPDE5-T7, or the empty vector pSNAP-tag (T7) as a control. When analyzed by immunoblot using a rabbit polyclonal antibody against human PDE5, a protein band migrating close to the predicted molecular weight of 93.7 kDa was detected only in the extract made from cells carrying pPDE5-T7 (Figure 2a, lane 4), but not in extract from cells carrying pSNAP-tag(T7) (Fig. 2a, lane 3). Coomassie blue staining of the gel showed that there was no obvious protein degradation in either sample (Figure 2a, lanes 1 and 2). These data showed that pPDE5-T7 expressed a feline protein that was specifically recognizable by an anti-human PDE5 antibody.

To confirm that the expressed protein was PDE5, a cGMP specific phosphodiesterase, we measured cGMP hydrolysis activity of the cell extracts in the presence or absence of a known PDE5 inhibitor, MY5445. As shown in Figure 2b, extracts from cells carrying pPDE5-T7 showed PDE activity toward cGMP. Furthermore, this PDE activity was inhibited by 30% when MY5445 was included in the reaction mixture. Thus, the isolated cDNA encodes a feline PDE5 allele and its translated protein product can be recognized by an anti-human PDE5 antibody.

### Expression of PDE5 and Detection of PDE Activity in Feline Cardiac Tissues

**Figure 3:**
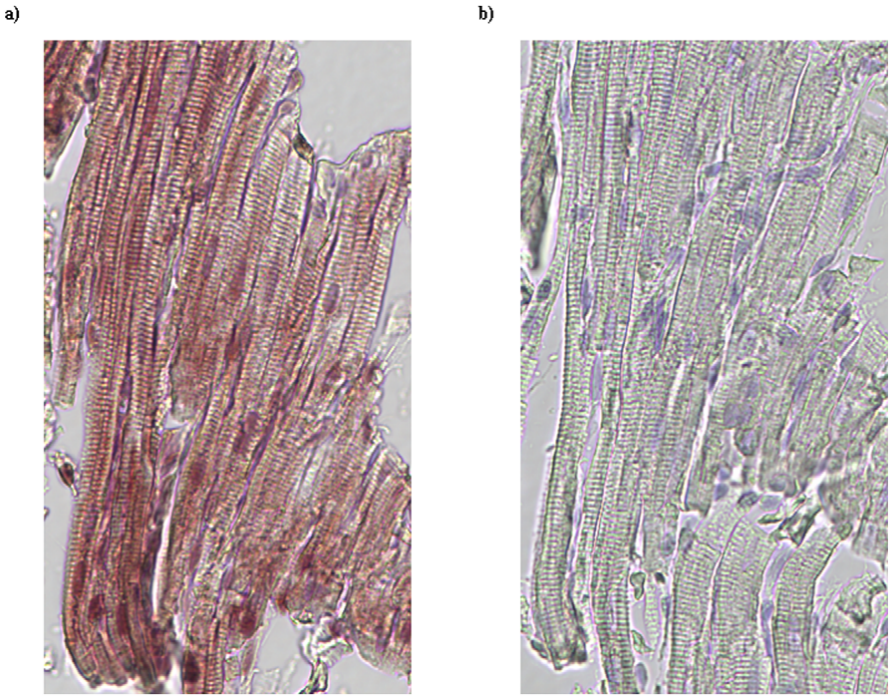
Detection of PDE5 expression in myocytes of feline RV by IHC staining. Consecutive paraffin embedded RV tissue sections from control feline hearts were used to examine PDE5 expression. The section in a) was incubated first with a rabbit polyclonal anti-PDE5 antibody followed by HRP conjugated anti-rabbit secondary antibody and then developed using ImmPACT NovaRED as a substrate to visualize the bonded antibodies. Section b) was processed in parallel with a) but omitted the primary anti-PDE5 antibody. Hematoxylin QS was used as nuclear counterstaining.

**Figure 4:**
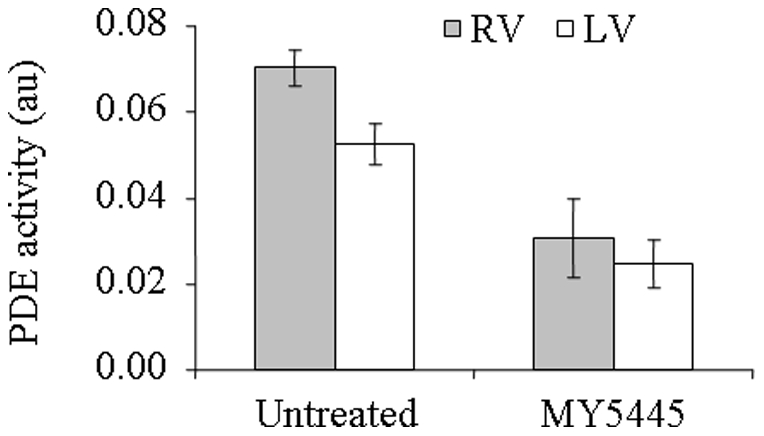
Detection of cGMP specific PDE activities in feline cardiac tissues. Tissue homogenates from RV and LV of control animals were prepared as described in Methods. cGMP specific PDE activity in these samples were measured in the presence or absence of 120 uM MY5445, a specific inhibitor of PDE5, as described in Methods. The error bars indicate standard deviation (n = 4).

To assess the presence of PDE5 in feline cardiac tissue, we carried out immunohistochemical (IHC) staining on paraffin sections of RV from the control group. As shown in Figure 3, a section incubated with antibody against PDE5 produced positive staining in cardiac myocytes with characteristic striation (Fig. 3A) while a consecutive section processed in the same manner but omitted the primary antibody did not display positive staining (Fig. 3b). This data demonstrated the presence of PDE5 in feline cardiac myocytes as previously observed in human specimens [12], [23]. To confirm the presence of PDE5 in these samples, we then measured PDE activity in whole cell extracts made from both RV and LV tissues of the same group of animals. Figure 4 showed that PDE activity was detected in both the RV and the LV tissue samples. Furthermore, addition of MY5445, a PDE5 inhibitor, led to a significant reduction of the PDE activity, by 56% in the RV and 53% in the LV. This result strongly suggested that a large portion of the measured PDE activity was that of PDE5, thus confirming the immunofluorescence data.

### Establishment of Feline Hypertrophy Models

Feline PA- and Ao-banded models were created to simulate the hypertrophy of the right ventricle (RV) or left ventricle (LV) associated with pulmonary or systemic hypertension, respectively. These models facilitated the study of PDE5 expression under the hypertrophy conditions. A total of 12 animals in 3 groups, i.e. Control, PA-banded and Ao-banded, were used. All tissue samples but one were collected after one month of banding. Tissue samples from the age-matched control group were collected at the same time. One PA-banded sample was collected after 2 month of banding. None of the animals included in these studies manifested overt severe failure with ascites, pulmonary edema, lethargy or cyanosis. Changes in cardiac morphology induced after one month of Ao-banding or PA-banding are presented in Table 1 along with analogous measurements in age-matched non-banded controls. Both Ao- and PA-banding resulted in significant increases in the heart weight to body weight ratio. In the Ao-banded animals at one-month, echocardiography revealed a substantial increase in the thickness of the intraventricular septum and the left ventricular posterior wall with smaller, but significant increases in right ventricular free wall thickness and left ventricular end-diastolic dimension. These changes were associated with a marked increase in the estimated LV mass and an insignificant decrease in left ventricular fractional shortening. In the PA-Banded animals at one-month, there was a substantial thickening of the right ventricular free wall, but no other significant changes. The data shows that increased resistance to ventricular outflow produced load-induced hypertrophy of the relevant chamber. Although we did not directly measure pulmonary artery pressures, the significant increase in right ventricular wall thickness observed in the animals with Ao-banding indirectly suggests that elevated pulmonary pressures were present in this model.

**Table 1:** Cardiac Morphology at 1 Month after Banding.

	HW/BW (mg/g)	RV th (mm)	IVS th (mm)	PW th (mm)	LVEDD (mm)	LV FS (mm)	LV Mass (mm)
**Controls (1 mo.)**	5.6±0.4	1.5±0.1	3.2±0.1	3.5±0.2	8.1±0.5	58±6	2.8±0.3
**Ao-Banded (1 mo.)**	11.9±0.4*	2.2±0.2*	6.4±0.2*	7.6±0.6*	10.1±0.4*	43±1	11.2±1.1*
**PA-Banded (1 mo.)**	9.1±0.7*	3.9±0.4*	4.3±0.5	4.2±0.4	7.4±0.3	44±4	3.6±0.5

Values are mean ± SEM.

Abbreviations: HW/BW-heart weight to body weight ratio; RV th-RV free wall thickness; IVS th-thickness of interventricular septum; PW th-thickness of left ventricular posterior wall; LVEDD-left ventricular end-diastolic dimension; LVFS-left ventricular fractional shortening; LV Mass-calculated left ventricular mass (see text for details).

*p<0.05 vs. Control.

### Altered PDE5 Expression Levels in the Hypertrophy Models

**Figure 5:**
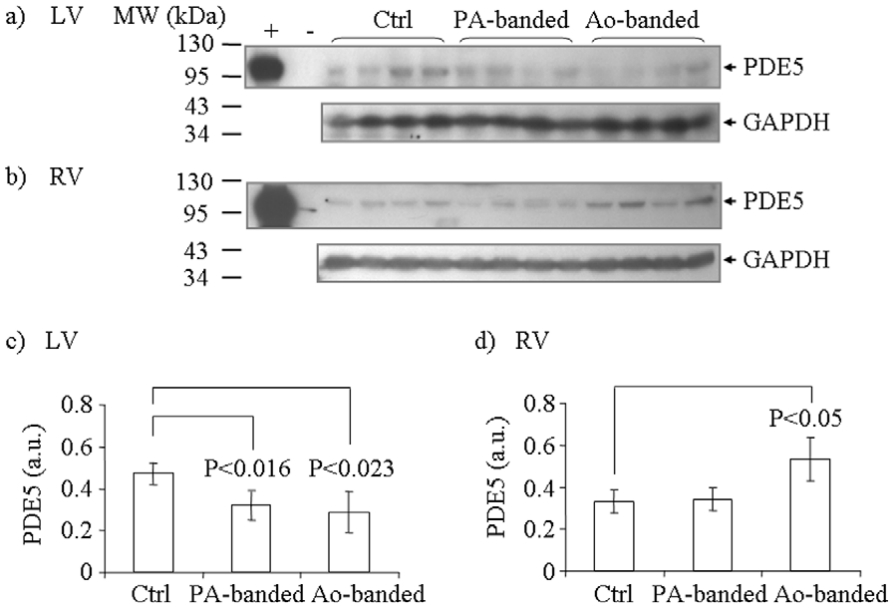
Determination of PDE5 expression in feline hypertrophy models. Tissue homogenates from LV and RV of control, PA-banded and Ao-banded animal models were analyzed for PDE5 expression by immunoblotting. (a) Homogenates from LV samples. (b) Homogenate from RV samples. Samples from control, PA-banded and Ao-banded groups were indicated in the figure. *E. coli* extracts from strains carrying pT7-PDE5 and pSNAP-tag(T7) were used as positive and negative controls (shown as+and -). GAPDH levels in the same set of tissue samples were also determined by immunoblotting, and used for normalization of PDE5 levels. (c) Normalized average PDE level in LV tissues from each group (n = 4). (d) Normalized average PDE level in RV tissues from each group. The error bars indicate standard deviations. Student t-test p values that show statistical significance are indicated in the figure.

To examine the expression level of PDE5, homogenates of RV and LV tissues from the control, PA- and Ao-banded animal groups were prepared and PDE5 protein content in these samples were assessed by immunoblotting, using the anti-human PDE5 antibody that recognized feline PDE5 expressed in E. coli (Figure 5a and 5b). GAPDH levels in these samples were also measured as an internal control of total protein content. The expression level of PDE5 in each sample, determined by densitometry, was then normalized to the GAPDH expression level, and the average expression level of each group (n = 4) was calculated (Figure 5c and 5d). As shown in Figure 5c and 5d, compared with control group, average PDE5 expression was reduced by 39% in the LV (p<0.023), but increased by 61% in the RV (p<0.05) of Ao-banded animals. Similarly, average PDE5 expression was reduced by 32% in the LV (p<0.016) of PA-banded animals. However, no statistically significant change in PDE5 expression in the RV of PA-banded animals was detected, in contrast to the RV of Ao-banded animals. These results showed that PDE5 expression responded differently in RV and LV to hypertrophy condition, and the response was also distinctive in the different hypertrophy models.

### Differential Expression of cGKI in Feline Hypertrophy Models

**Figure 6:**
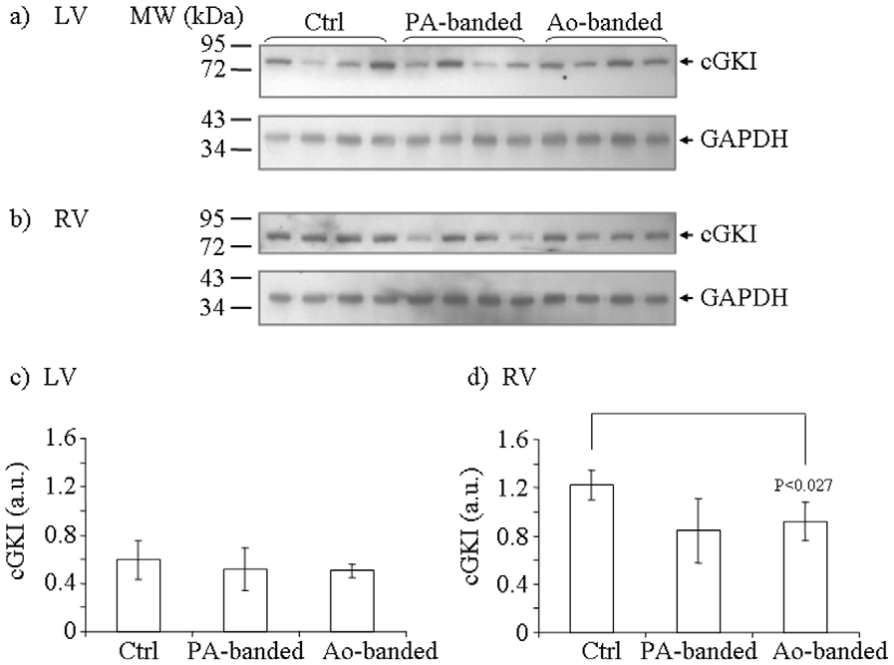
cGKI expression in feline hypertrophy models. Tissue homogenates from LV (a) and RV (b) of control, PA-banded and Ao-banded animal models were analyzed for cGKI expression by immunoblotting, as described in Figure 5. GAPDH levels in the same set of samples were used to normalize the cGKI levels. The normalized average cGKI levels in each group (n = 4) are shown in (c) for LV and (d) in RV. The error bars indicate standard deviations. Student t-test p values that show statistical significance are indicated in the figure.

Recognizing that cyclic GMP dependent kinase I (cGKI) is a major downstream effector of cGMP, we examined the expression levels of cGKI in the same sets of tissue samples by immunoblotting, using an anti-cGKI antibody (Figure 6a and 6b). After normalizing to GAPDH, the average expression level of each group (n = 4) was calculated and shown in Figure 6c and 6d. In Ao-banded animals, we observed no detectable change of cGKI abundance in the LV, but a 32% reduction of cGKI was observed in the RV (p<0.027), compared to the control group. In the PA-banded group, no statistically significant change in cGKI expression level was observed in either the LV or the RV. Our data thus indicate that cGKI response to Ao-banding is also different in RV and LV, with a reciprocal relationship between PDE5 (up) and cGKI (down) only observed in the RV. Furthermore, cGKI expression and PDE5 expression patterns appear uncoupled in PA-banded animals.

## Discussion

In this study, we investigated the regulation of PDE5 expression in LV and RV of Ao- and PA-banded feline hypertrophy models. A cDNA fragment encoding the feline PDE5 protein was isolated and sequenced. The protein shared 98% sequence identity with its human counterpart and was highly conserved structurally. The difference between the feline and human PDE5 was primarily in the 5′- end region, while the regions corresponding to the regulatory and catalytic domains of the human PDE5 were nearly identical. The feline PDE5 cDNA was cloned and expressed in *E. coli* to allow the identification of antibodies that specifically recognize the feline PDE5 protein. This permitted elucidation of chamber-specific responses in the regulation of PDE5 expression to pressure overload.

In humans, there are three PDE5 isozymes, A1, A2, and A3, due to alternative splicing of the same transcript [5]. The cloned putative feline PDE5 is most similar to the human PDE5-A3, which has the shortest N-terminal region (see Figure 1). Several studies have shown that the splicing process for PDE5 could be tissue type dependent [5], [24]. In this regard, our data indicate that feline smooth muscle cells express the A3 variant. Expression of the putative feline PDE5 in *E. coli* allowed us to measure its enzyme activity and to screen for antibodies that recognize this protein. By immunoblotting, a polyclonal antibody against the human PDE5 was shown to recognize the putative feline PDE5 protein specifically. It is worth noting that since this antibody cross-reacted with the shortest form of PDE5, A3, it is unlikely to distinguish among the three different variants. Therefore, the protein we examined in feline cardiac tissue with this antibody could be any isoform, or a combination of multiple isoforms of PDE5.

MY5445 is a PDE5 specific inhibitor used in many studies to selectively inhibit the enzyme [25], [26], [27]. However, MY5445 has reduced potency in inhibiting cGMP-hydrolysis in non-mammalian system for reasons that remain unclear [28]. In our study, a significant but incomplete inhibition of enzyme activity was observed when extracts made from *E. coli* expressing the feline PDE5 cDNA were used. The level of inhibition was almost doubled when feline cardiac tissue extracts were used instead of bacterial extracts. This suggests that proper post-translational modification and folding of PDE5 is important for the inhibitor action. Phosphorylation of PDE5 was reported to increase binding affinity of Tadalafil to the catalytic site, which could lead to increased potency of the inhibitor [29]. The N-terminal phosphorylation site in PDE5 is conserved from human to feline. However, it is unlikely that the protein expressed in *E. coli* will be properly phosphorylated. Thus, the lack of adequate post-translational modification, or proper folding, both could contribute to the reduced inhibition efficiency of MY5445 against the *E. coli* expressed PDE5. Nevertheless, the near identical sequence to human PDE5, the cross-reactivity to the anti-human PDE5 antibody and the presence of MY5445 inhibitable phosphodiesterase activity against cGMP demonstrated that the cloned cDNA encodes feline PDE5.

Despite the great versatility and utility of rodent models, cardiac excitation-contraction coupling and contractile reserve dynamics in rodents differ from those of large mammals [30]. In addition, cardiovascular and total body metabolism is also different between small and large mammals [31]. Furthermore, small molecules may not achieve the same effect when tested in different species [32], [33]. Most relevant for our studies, we observed differences in our comparison of PDE5 cDNA primary structures across species. In all mentioned aspects, feline is closer than rodent to human. In fact, Vandeput et al have shown differences in Sildenfil inhibition of cGMP-hydrolytic activity between human and mouse that could be due to interactions of other members in the cGMP specific PDE sub-family [33]. Based on these considerations, we feel there are advantages for using larger mammals, including felines, to gain insights into pathologic myocardial adaptations in humans.

We studied PDE5 expression under hypertrophic conditions, and observed chamber-dependent differential expression of PDE5. Our finding of reduced PDE5 levels in the LV of the Ao-banded animals is consistent with the result by Senzaki et al using a tachypacing canine model to induce LV failure [34]. The somewhat surprising findings of increased PDE5 expression in the RV of Ao-banded animals, coupled with the observation that in PA-banded animals PDE5 expression was reduced in the LV but unchanged in the RV, suggests that hemodynamic overload could induce chamber-dependent factors in regulating PDE5 expression in the heart. These chamber-dependent factors, upon sensing the stress due to hemodynamic overload, could lead to differential responses from the downstream pathways in the RV and LV. Although decreases in cGKI, a downstream effector of cGMP, were observed in association with increases in RV PDE5 in the PA-banding model, decreases in PDE5 in the LV were not paralleled by increases in cGKI. Dissociation of RV and LV PDE5 expression and an inconsistent relationship between PDE5 and cGKI expression provided further evidence that the signal transduction pathways involving PDE5 during cardiac hypertrophy could be regulated differently in the two chambers, as recently suggested by others commenting on incongruent responses to in vivo PDE5 inhibition [16], [35]. Moreover, Schafer et al. reported that beneficial effects of PDE5 inhibition was dependent on whether the RV remodeling was achieved through monocrotaline-induced pulmonary hypertension or PA banding, with the latter not benefiting from the inhibition of PDE5 [14]. Other studies also indicated that beneficial effects of PDE5 inhibition were dependent on the pressure-overload magnitude, suggesting the possibility that differences in chamber geometry may modulate the effects of a fixed resistance [15]. Conceivably, differences in the embryologic origins of the RV and LV and/or chamber-specific distinctions in the isoforms of functionally important transcription factors [36] could produce differences in the RV and LV responses to seemingly equivalent pathological stress. Regardless of the mechanism, our findings contribute to a growing body of evidence demonstrating that the right and left ventricles, with their distinct developmental origins, may adopt different mechanisms to adapt to pathological stress, including hemodynamic overload. The differential regulation of PDE5 expression in response to hemodynamic overload is likely to be of important physiological significance, although the exact nature is not clear at this time. Further understanding of the detailed molecular mechanism of the chamber specific regulation of PDE5 expression and its functional consequences will help us understand the physiological significance.

In conclusion, the study of PDE5 and cGKI expression in the RV and LV after Ao and PA banding in felines indicated that PDE5 protein expression in the two chambers responded differently, suggesting heterogeneity in the regulation of cGMP/cGKI in hypertrophy. Accordingly, the pathogenic role of changes in PDE5 expression and the impact of therapeutic PDE5 inhibition may well differ for right and left ventricles and with different disease etiologies.

## Materials and Methods

### Ethics Statement

The investigation conformed to the *Guide of the Care and Use of Laboratory Animals* published by the US National Institutes of Health (NIH Publication No. 85-23, revised 1996), and all protocols were approved by the appropriate institutional animal care and use committees.

### Chemicals and Reagents

All chemicals were purchased from Sigma-Aldrich (St. Louis, MO, USA) unless otherwise indicated. Rabbit-polyclonal antibody against human PDE5 was from Cell Signaling (Danvers, MA) and rabbit-polyclonal antibodies against cGKI and GAPDH were from Santa Cruz Biotechnology (Santa Cruz, CA). All the secondary antibodies were from Jackson ImmunoResearch Laboratories, Inc. (West Grove, PA). MY5445, 1-(3-chlorophenylamino)-4-phenylphthalazine, was purchased from Santa Cruz Biotechnology.

### Isolation of Aortic Smooth Muscle Cells and cDNA Library Preparation

Aortic smooth muscle cells were isolated by an enzyme digestion method [37]. Briefly, about 2 cm feline aorta was removed and trimmed. The tissue was then minced to small pieces (about 2 mm/side) before subjecting it to collagenase type II (Worthington Biochemical Corp., Lakewood, NJ) digestion at 180 U/ml in DMEM/10% FBS at 37°C under sterile conditions. After 4 hours, dissociated cells were removed and harvested by centrifugation at 500× g. The cells were immediately seeded and cultured in DMEM/10% FBS under 5% CO2. The cell type was subsequently confirmed by immunofluorescence using a mouse monoclonal antibody against smooth muscle alpha actin (Sigma-Aldrich). A cDNA library was generated by first isolating total RNA from these cells using TRIzol (Invitrogen, Carlsbad, CA), then by reverse transcription using Superscript II (Invitrogen) according to the manufacturer's instructions.

### Isolation of PDE5 cDNA of Feline Origin

The internal and 3′- sequence of PDE5 cDNA were obtained from the smooth muscle cDNA library by PCR, using primers: forward 5′-TCAATGCAGAAGTTGACCAA and reverse 5′-GTTTCATCTGGAAGTTCTGC for the internal sequence and forward 5′-GTCACACTGGAGGTTCTGTC and reverse 5′-AAAATACAGCAGTGGCAAAG for the 3′- end sequence. The 5′- end of the cDNA was obtained by rapid amplification of cDNA ends (RACE), using SMART RACE cDNA Amplification kit (Clontech, Mountain View, CA) according to the manufacturer's instructions. Specifically, total RNA isolated from the smooth muscle cells were used for generating a RACE library. To amplify cDNA fragments from the RACE library corresponding to the 5′-end of PDE5, a universal primer provided in the kit served as the forward primer and a gene specific primer 5′-GAATGTCCCACCATTTCCCG served as the reverse primer. Three fragments with the length of ∼400, 1024 and ∼1050 bases were obtained and all three were sequenced. Based on the obtained sequence data, primers were designed to amplify the full-length cDNA (forward 5′-AAGCAGTGGTATCAACGCAGAGT and reverse 5′-AAAATACAGCAGTGGCAAAG) that spanned the open reading frame (ORF). The sequence of all the fragments were used to assemble contigs that represents an entire PDE5 ORF and part of the 5′ and 3′ untranslated region.

### Bacterial Expression of Feline PDE5 cDNA and Preparation of Cell Extracts

A DNA fragment containing the complete ORF of PDE5 was obtained by PCR using primers: forward 5′-GAAATGGTCAATGCCTGGTTTG and reverse 5′- ACTCAGTTCCGCTTGGCCTG with the full length cDNA from 2.3 as the template. The PCR product was cloned into a TA cloning vector, pGEMT-easy (Promega Corporation, Madison, WI). Several clones were sequenced to select ones that were devoid of mutations introduced during the PCR. A NdeI/EcoRI fragment of a mutation-free clone was then excised for sub-cloning into an *E. coli* expression vector pSNAP-tag(T7) (New England Biolabs, Ipswich, MA) at NdeI and EcoRI site. The vector was transformed into *E. coli* expression strain BL21-AI (Invitrogen). Expression of PDE5 was induced by adding arabinose to 0.1% final concentration at 37°C when the *E. coli* culture density reached 0.3 O.D and the induction duration was 3 hr. The cells were harvested and lysed by sonication in 10 mM Tris buffered saline, pH 7.4 (TBS) containing protease inhibitors (1 mM PMSF/protease inhibitor cocktail (Sigma-Aldrich)). Cell debris was removed by centrifugation and glycerol was added to the extract at a final concentration of 15%. The extracts were then stored at −80°C. Protein contents in the samples were quantified using BCA Protein Assay (Thermo Fisher Scientific Inc., Rockford, IL) and integrities were verified by Coomassie G-250 staining. Briefly, after electrophoresis, the gel was rinsed in water before incubating in a staining solution (0.1% Coomassie Blue R-250/10% methanol/10% acetic acid) for 1 hr. The gel was then de-stained in a solution containing 10% acidic acid/10% methanol for 2 hours.

### Measurement of PDE Activity

PDE activity of the *E. coli* cell extracts was measured using a cyclic nucleotide phosphodiesterase assay kit (Enzo Life Sciences International, Inc., Plymouth Meeting, PA) according to the manufacturer's instructions. In each reaction, an extract sample containing about 8 ug of total *E. coli* protein was used for the assay. To measure enzyme activity of feline cardiac tissue samples, extracts were first made by homogenizing cardiac tissue samples in TBS containing protease inhibitors (1 mM PMSF/protease inhibitor cocktail (Sigma-Aldrich)). After removing debris by centrifugation at 10,000× g, glycerol was added to a final concentration of 15%. The extracts were then aliquoted and stored at −80°C until assaying. Tissue extracts containing 15 µg of total protein were used in assay reaction. MY5445 was added to a final concentration of 120 µM, as a PDE inhibitor, when necessary.

### Feline Model for Cardiac Hypertrophy

The animal models were created using a previously reported protocol [38]. Briefly, after anesthesia induction with ketamine (50 mg/kg) and acepromazine (0.5 mg/kg), young cats (8–10 weeks old) were intubated and ventilated with room air and 1% isoflurane to maintain a surgical plane of anesthesia. Using aseptic technique, a right thoracotomy and pericardiotomy were performed and the pulmonary artery was dissected free from the aorta. A 3.0 mm (internal diameter) clip was then placed around the proximal pulmonary artery (PA) for the PA banding model or around the ascending aorta (Ao) for the Ao banding model. The lungs were fully expanded and the chest was closed. We used only age-matched non-operated animals as controls in this study.

### Echocardiography

Two-dimensional and M-mode echocardiography was performed using an Acuson Cypress hand-held echocardiographic platform with a 7V3c transducer. All animals were sedated with an intramuscular injection of Ketamine (50 mg/kg) and Acepromazine (0.5 mg/kg) prior to echocardiography. Short-axis M-mode displays were used to measure the thickness of the right ventricular free wall, interventricular septum (IVS), and left ventricular posterior wall (PW) and the left ventricular dimensions at end-diastole (LVEDD) and end-systole (LVESD). All measurements were made in triplicate and averaged. Left ventricular fractional shortening (LV FS) was calculated as:
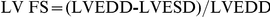
Left ventricular mass (grams) was estimated using the formula validated by Devereux *et al* [39].
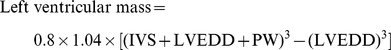



### Immunohistochemistry and Immunoblotting (IB)

Isolated right ventricular tissues from control felines were immediately fixed in 4% formaldehyde followed by paraffin-embedding. Immunohistochemical staining was carried out using a rabbit anti-human PDE5 antibody and the ImmPRESS Reagent anti-rabbit Ig (Vector Laboratories, Burlingame, CA) according to the manufacturer's instructions. Briefly, sections were de-paraffinized and re-hydrated. A citrate buffer, 10 mM citric acid, 0.05% Tween 20, pH 6.0, was used to unmask antigens at 100°C for 20′ and the endogenous peroxides was quenched by incubating the sections in PBS/2% H_2_O_2_ for 15′ at RT. After blocking with 2.5% normal horse blocking serum, PDE5 expression in the tissue was detected with the anti-human PDE5 and HRP-conjugated secondary antibodies. ImmPACT Nova RED (Vector Laboratories) was used to visualize PDE5 staining and hematoxylin QS (Vector Laboratories) was used for nuclear counterstaining. Background controls employed only the secondary antibody. The stained sections were mounted in CitraMount Medium (Polysciences, Inc., PA). Previously described protocol for IB was used [40]. Briefly, flash-frozen RV and LV tissues were homogenized in PBS/protease inhibitors (1 mM PMSF/protease inhibitor cocktail (Sigma-Aldrich)) and debris removed by centrifugation. Tissue extracts were then mixed with Lammli buffer and loaded onto an 8% polyacrylamide gel. After electrophoresis, the proteins were transferred to a PVDF membrane and blocked with 5% non-fat dry milk. The membrane was then incubated with a rabbit polyclonal anti-PDE5 or anti-GAPDH antibodies followed by incubation with a secondary HRP-conjugated goat anti-rabbit secondary antibody. The membrane was washed with PBS/0.1% Tween-20 between each incubation step. Secondary antibodies bound to the membranes were detected by ECL substrate and visualized after exposure to x-ray films.

### Statistical Analysis

A one-way between subjects ANOVA was conducted to compare the Control, PA-Banded and Ao-Banded subjects. Post hoc comparisons using the Bonferroni-Holm test were used to examine pairwise comparisons.
